# Complete genome sequence of *Cryptobacterium curtum* type strain (12-3^T^)

**DOI:** 10.4056/sigs.12260

**Published:** 2009-09-24

**Authors:** Konstantinos Mavrommatis, Rüdiger Pukall, Christine Rohde, Feng Chen, David Sims, Thomas Brettin, Cheryl Kuske, John C. Detter, Cliff Han, Alla Lapidus, Alex Copeland, Tijana Glavina Del Rio, Matt Nolan, Susan Lucas, Hope Tice, Jan-Fang Cheng, David Bruce, Lynne Goodwin, Sam Pitluck, Galina Ovchinnikova, Amrita Pati, Natalia Ivanova, Amy Chen, Krishna Palaniappan, Patrick Chain, Patrik D'haeseleer, Markus Göker, Jim Bristow, Jonathan A. Eisen, Victor Markowitz, Philip Hugenholtz, Manfred Rohde, Hans-Peter Klenk, Nikos C. Kyrpides

**Affiliations:** 1 DOE Joint Genome Institute, Walnut Creek, California, USA; 2 DSMZ - German Collection of Microorganisms and Cell Cultures GmbH, Braunschweig, Germany; 3 Los Alamos National Laboratory, Bioscience Division, Los Alamos, New Mexico, USA; 4 Biological Data Management and Technology Center, Lawrence Berkeley National Laboratory, Berkeley, California, USA; 5 Lawrence Livermore National Laboratory, Livermore, California, USA; 6 University of California Davis Genome Center, Davis, California, USA; 7 HZI - Helmholtz Centre for Infection Research, Braunschweig, Germany

**Keywords:** oral infections, opportunistic pathogenic, periodontitis, non-spore-former, anaerobic, asaccharolytic, *Coriobacteriaceae*

## Abstract

*Cryptobacterium curtum* Nakazawa *et* *al*. 1999 is the type species of the genus, and is of phylogenetic interest because of its very distant and isolated position within the family *Coriobacteriaceae*. *C. curtum* is an asaccharolytic, opportunistic pathogen with a typical occurrence in the oral cavity, involved in dental and oral infections like periodontitis, inflammations and abscesses. Here we describe the features of this organism, together with the complete genome sequence, and annotation. This is the first complete genome sequence of the actinobacterial family *Coriobacteriaceae*, and this 1,617,804 bp long single replicon genome with its 1364 protein-coding and 58 RNA genes is part of the *** G****enomic* *** E****ncyclopedia of* *** B****acteria and* *** A****rchaea * project.

## Introduction

Strain 12-3^T^ (= DSM 15641 = ATCC 700683 = CCUG 43107) is the type strain of *Cryptobacterium curtum*, which is the sole species within the genus *Cryptobacterium* [1]. *C. curtum* was described by Nakazawa *et* *al*. in 1999 [1]. The organism is of significant interest because of its position in the tree of life where it was initially wrongly placed close to *Eubacterium* (*Firmicutes*) to be then relocated in the phylum *Actinobacteria,* close to the *Coriobacteriaceae* [1]. Here we present a summary classification and a set of features for *C. curtum* 12-3^T^, together with the description of the complete genomic sequencing and annotation.

## Classification and features

**Figure 1:**
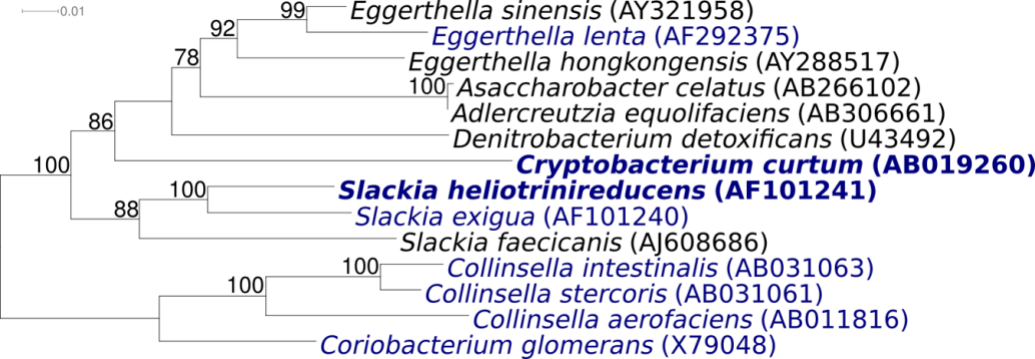
Phylogenetic tree of *C. curtum* 12-3^T^ and most type strains of the family *Coriobacteriaceae*, inferred from 1422 aligned 16S rRNA characters [3,4] under the maximum likelihood criterion [5]. The tree was rooted with type strains of the genera *Collinsella* and *Coriobacterium*. The branches are scaled in terms of the expected number of substitutions per site. Numbers above branches are support values from 1000 bootstrap replicates if larger than 60%. Strains with a genome sequencing project registered in GOLD [6] are printed in blue; published genomes in bold, including two of which are reported in this issue of *SIGS* [7,8]

The type strain 12-3^T^ and a second strain of the species, KV43-B, both classified in *C. curtum* were isolated from a periodontal pocket sample of an adult patient and from necrotic dental pulp, respectively [1]. *C. curtum* can also be isolated from human oral and dental infections like pulpal inflammations, advanced caries [1], dental abscesses or periodontitis [2]. 16S rRNA gene sequence analysis revealed that the two isolates represent a distinct lineage within the family *Coriobacteriaceae*, between the neighboring genera *Eggerthella* and *Slackia* (Figure 1). No significant matches with any 16S rRNA sequences from environmental genomic samples and surveys are reported at the NCBI BLAST server (February 2009).

**Figure 2:**
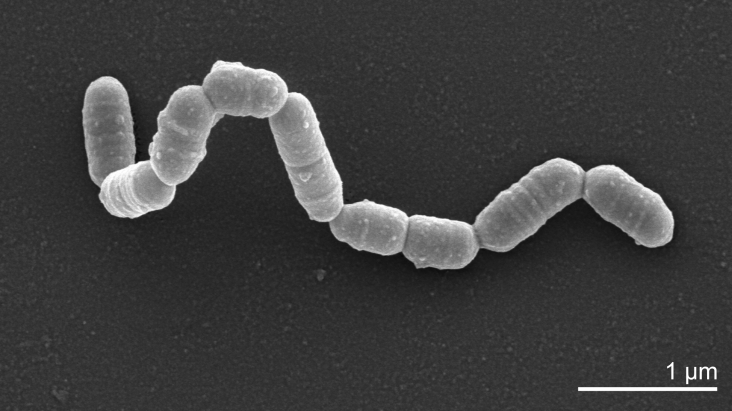
Scanning electron micrograph of *C. curtum* 12-3 ^T^

The very short and non-motile rods form tiny translucent colonies of less than 1 mm in diameter on BHI-blood agar without hemolysis after prolonged incubation under strictly anaerobic conditions (Table 1). Transmission electron micrographs of ultrathin sections of *C. curtum* 12-3^T^ showed a single-layered Gram-positive cell wall of approximately 10 nm thickness (Figure 2) [1]. Carbohydrates are not metabolized; the species is asaccharolytic [1]. *C. curtum* is non-reactive in most biochemical tests. The human oral cavity contains arginine and other amino acids and oligopeptides due to proteinase and peptidase activities. *C. curtum* degrades arginine through arginine deiminase pathway [15]. Like *Slackia exigua*, a closely related species, these bacteria are very difficult to cultivate. Optimal doubling time is 12 hours [15]. There are no chemotaxonomic data available to *C. curtium* strain 12-3^T^.

**Table 1:** Classification and general features of *C. curtum* 12-3^T^ according to the MIGS recommendations [9]

**MIGS ID**	**Property**	**Term**	**Evidence code**
	Current classification	Domain *Bacteria*	TAS [10]
		Phylum *Actinobacteria*	TAS [11]
		Class *Actinobacteria*	TAS [12]
		Order *Coriobacteriales*	TAS [12]
		Family *Coriobacteriaceae*	TAS [12]
		Genus *Cryptobacterium*	TAS [1]
		Species *Cryptobacterium curtum*	TAS [1]
		Type strain 12-3	TAS [1]
	Gram stain	positive	TAS [1]
	Cell shape	very short rods	TAS [1]
	Motility	nonmotile	TAS [1]
	Sporulation	non-sporulating	TAS [1]
	Temperature range	mesophile	TAS [1]
	Optimum temperature	37°C	NAS
	Salinity	normal	TAS [1]
MIGS-22	Oxygen requirement	obligate anaerobic	TAS [1]
	Carbon source	asaccharolytic	TAS [1]
	Energy source	arginine, lysine	NAS
MIGS-6	Habitat	human oral microflora	TAS [1]
MIGS-15	Biotic relationship	free living, growth on enzymatic degradation products of inflamed tissues	NAS
MIGS-14	Pathogenicity	periodontal infections	TAS [1]
	Biosafety level	1 (+)	TAS [13]
	Isolation	infected human oral cavity	TAS [1]
MIGS-4	Geographic location	not reported	NAS
MIGS-5	Sample collection time	about 1995	TAS [1]
MIGS-4.1 MIGS-4.2	Latitude – Longitude	not reported	
MIGS-4.3	Depth	not reported	
MIGS-4.4	Altitude	not reported	

Evidence codes - IDA: Inferred from Direct Assay (first time in publication); TAS: Traceable Author Statement (i.e., a direct report exists in the literature); NAS: Non-traceable Author Statement (i.e., not directly observed for the living, isolated sample, but based on a generally accepted property for the species, or anecdotal evidence). These evidence codes are from the Gene Ontology project [14]. If the evidence code is IDA, then the property was directly observed for a live isolate by one of the authors, or an expert or reputable institution mentioned in the acknowledgements.

Figure 1 shows the phylogenetic neighborhood of *C. curtum* strain 12-3^T^ in a 16S rRNA based tree. Analysis of the three 16S rRNA gene sequences in the genome of strain 12-3^T^ indicated that the genes differ by at most one nucleotide from each other, but differ by 15 nucleotides and eight ambiguities (1.1%) from the previously published 16S rRNA sequence generated from DSM 15641 (AB019260). The higher sequence coverage and overall improved level of sequence quality in whole-genome sequences, as compared to ordinary gene sequences, implies that the significant differences between the genome data and the reported 16S rRNA gene sequence might be due to sequencing errors in the previously reported sequence data.

## Genome sequencing and annotation

### Genome project history

This organism was selected for sequencing on the basis of each phylogenetic position, and is part of the *** G****enomic* *** E****ncyclopedia of* *** B****acteria and* *** A****rchaea * project [16]. The genome project is deposited in the Genome OnLine Database [6] and the complete genome sequence in GenBank. Sequencing, finishing and annotation were performed by the DOE Joint Genome Institute (JGI). A summary of the project information is shown in Table 2.

**Table 2:** Genome sequencing project information

**MIGS ID**	**Property**	**Term**
MIGS-31	Finishing quality	Finished
MIGS-28	Libraries used	Three genomic libraries: two Sanger libraries - 8 kb pMCL200 and fosmid pcc1Fos - and one 454 pyrosequence standard library
MIGS-29	Sequencing platforms	ABI3730, 454 GS FLX
MIGS-31.2	Sequencing coverage	12.9× Sanger; 20× pyrosequence
MIGS-30	Assemblers	Newbler version 1.1.02.15, phrap
MIGS-32	Gene calling method	Genemark 4.6b, tRNAScan-SE-1.23, infernal 0.81, GenePRIMP
	INSDC / Genbank ID	CP001682
	Genbank Date of Release	August 26, 2009
	GOLD ID	Gc01086
	NCBI Project ID	20739
	Database: IMG-GEBA	2500901758
MIGS-13	Source material identifier	DSM 15641
	Project relevance	Tree of Life, GEBA

### Growth conditions and DNA isolation

*C. curtum* strain 12-3^T^, DSM 15641, was grown anaerobically in DSMZ medium 78 (Chopped Meat Medium) [17], supplemented with 1 g/l arginine, at 37°C. DNA was isolated from 1-1.5 g of cell paste using Qiagen Genomic 500 DNA Kit (Qiagen, Hilden, Germany) with protocol modification st/FT [16] for cell lysis.

### Genome sequencing and assembly

The genome was sequenced using a combination of Sanger and 454 sequencing platforms. All general aspects of library construction and sequencing performed at the JGI can be found at http://www.jgi.doe.gov/. 454 Pyrosequencing reads were assembled using the Newbler assembler version 1.1.02.15 (Roche). Large Newbler contigs were broken into 1,799 overlapping fragments of 1000bp and entered into assembly as pseudo-reads. The sequences were assigned quality scores based on Newbler consensus q-scores with modifications to account for overlap redundancy and to adjust inflated q-scores. A hybrid 454/Sanger assembly was made using the parallel phrap assembler (High Performance Software, LLC). Possible mis-assemblies were corrected with Dupfinisher [18] or transposon bombing of bridging clones (Epicentre Biotechnologies, Madison, WI). Gaps between contigs were closed by editing in Consed, custom primer walk or PCR amplification. 47 Sanger finishing reads were produced to close gaps, to resolve repetitive regions, and to raise the quality of the finished sequence. The error rate of the completed genome sequence is less than 1 in 100,000. Together all sequence types provided 32.9x coverage of the genome.

### Genome annotation

Genes were identified using GeneMark [19] as part of the genome annotation pipeline in the Integrated Microbial Genomes Expert Review (IMG-ER) system [20], followed by a round of manual curation using the JGI GenePRIMP pipeline [21]. The predicted CDSs were translated and used to search the National Center for Biotechnology Information (NCBI) nonredundant database, UniProt, TIGRFam, Pfam, PRIAM, KEGG, COG, and InterPro databases. The tRNAScanSE tool [22] was used to find tRNA genes, whereas ribosomal RNAs were found by using the tool RNAmmer [23]. Other non coding RNAs were identified by searching the genome for the Rfam profiles using INFERNAL (v0.81) [24]. Additional gene prediction analysis and manual functional annotation was performed within the Integrated Microbial Genomes (IMG) platform (http://img.jgi.doe.gov) [25].

### Metabolic network analysis

The metabolic Pathway/Genome Database (PGDB) was computationally generated using Pathway Tools software version 12.5 [26] and MetaCyc version 12.5 [27], based on annotated EC numbers and a customized enzyme name mapping file. It has undergone no subsequent manual curation and may contain errors, similar to a Tier 3 BioCyc PGDB [28].

### Genome properties

**Figure 3:**
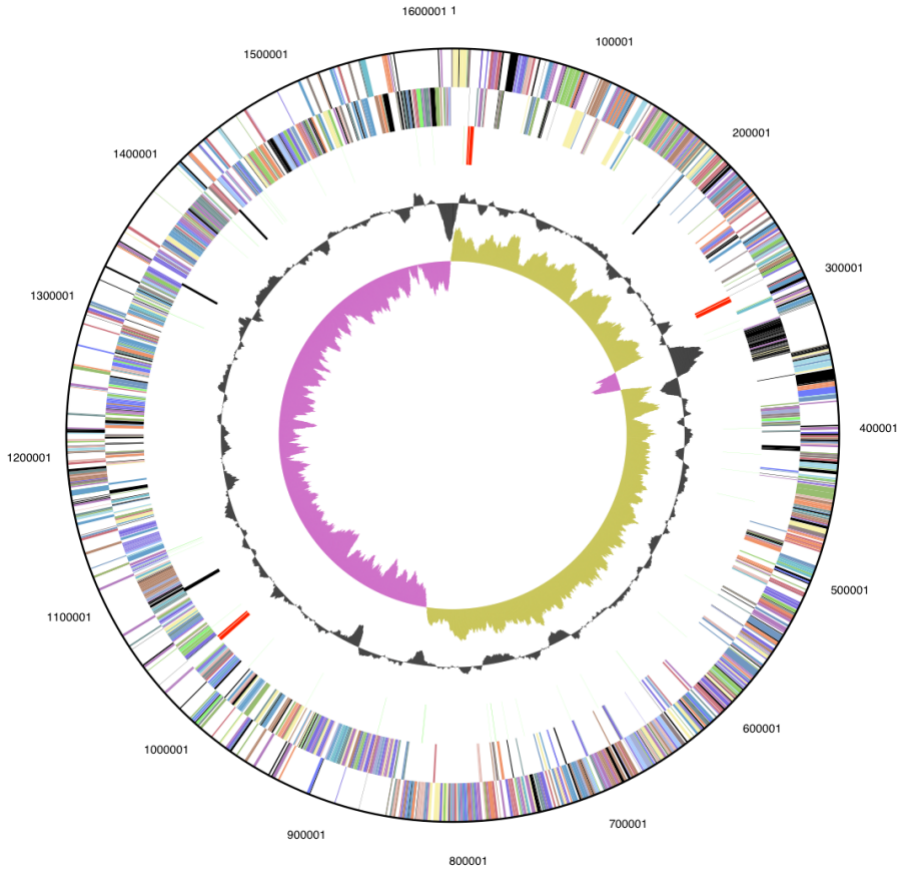
Graphical circular map of the genome. From outside to the center: Genes on forward strand (color by COG categories), Genes on reverse strand (color by COG categories), RNA genes (tRNAs green, rRNAs red, other RNAs black), GC content, GC skew.

**Figure 4:**
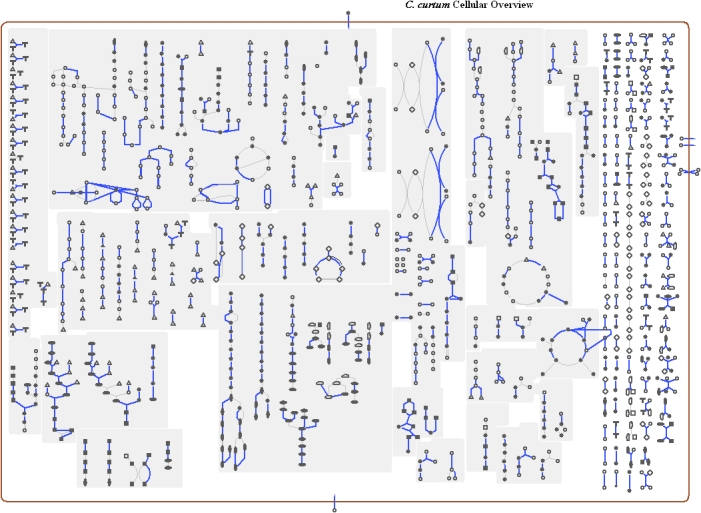
Schematic cellular overview diagram of all pathways of *C. curtum* 12-3^T^. Nodes represent metabolites, with shape indicating class of metabolite. Lines represent reactions.

The genome is 1,617,804 bp long and comprises one main circular chromosome with a 50.9% GC content (Table 3 and Figure 3). Of the 1422 genes predicted, 1364 were protein coding genes, and 58 RNAs. A total of 7 pseudogenes were also identified. Among the majority of protein coding genes (78.5%) were assigned with a putative function while the remaining were annotated as hypothetical proteins. The properties and the statistics of the genome are summarized in Table 3. The distribution of genes into COG functional categories is presented in Table 4, and a cellular overview diagram is presented in Figure 4, followed by a summary of metabolic network statistics shown in Table 5.

**Table 3:** Genome Statistics

**Attribute**	**Value**	**% of Total**
Genome size (bp)	1,617,804	
DNA Coding region (bp)	1,439,290	88.97%
DNA G+C content (bp)	823,649	50.91%
Number of replicons	1	
Extrachromosomal elements	0	
Total genes	1425	100.00%
RNA genes	58	2.37%
rRNA operons	3	
Protein-coding genes	1364	95.92%
Pseudo genes	7	0.49%
Genes with function prediction	1117	78.55%
Genes in paralog clusters	77	5.41%
Genes assigned to COGs	1103	77.57%
Genes assigned Pfam domains	1104	77.64%
Genes with signal peptides	276	19.37%
Genes with transmembrane helices	206	14.46%
CRISPR repeats	0	

**Table 4:** Number of genes associated with the general COG functional categories

Code	Value	%		Description	
J	128	9.4	Translation, ribosomal structure and biogenesis	
A	1	0.1	RNA processing and modification	
K	94	6.9	Transcription	
L	74	5.5	Replication, recombination and repair	
B	1	0.1	Chromatin structure and dynamics	
D	15	1.1	Cell cycle control, mitosis and meiosis	
Y	0	0.0	Nuclear structure	
V	20	1.5	Defense mechanisms	
T	64	4.7	Signal transduction mechanisms	
M	70	5.1	Cell wall/membrane biogenesis	
N	1	0.1	Cell motility	
Z	1	0.1	Cytoskeleton	
W	0	0.0	Extracellular structures	
U	20	1.5	Intracellular trafficking and secretion	
O	55	4.0	Posttranslational modification, protein turnover, chaperones	
C	100	7.3	Energy production and conversion	
G	41	3.0	Carbohydrate transport and metabolism	
E	96	7.0	Amino acid transport and metabolism	
F	47	3.4	Nucleotide transport and metabolism	
H	69	5.1	Coenzyme transport and metabolism	
I	39	2.9	Lipid transport and metabolism	
P	70	5.1	Inorganic ion transport and metabolism	
Q	9	0.7	Secondary metabolites biosynthesis, transport and catabolism	
R	119	8.7	General function prediction only	
S	81	5.9	Function unknown	
-	261	19.1	Not in COGs	

**Table 5:** Metabolic Network Statistics

**Attribute**	Value
Total genes	1422
Enzymes	316
Enzymatic reactions	606
Metabolic pathways	115
Metabolites	506
